# Axillary evaluation in women with elevated body mass index: evidence, challenges, and perspectives

**DOI:** 10.1590/0100-3984.2025.58.e11

**Published:** 2025-12-22

**Authors:** Renato Leme de Moura Ribeiro, Erica Elisangela Françolin Federicci

**Affiliations:** 1 Department of Radiology, Breast Imaging Group, Hospital Israelita Albert Einstein, São Paulo, SP, Brazil

A precise assessment of axillary lymph node status remains a cornerstone of staging and therapeutic planning in early-stage breast cancer. Axillary involvement is one of the most important prognostic factors, guiding surgical and systemic treatment strategies. In recent decades, surgical management of the axilla has evolved from complete dissection to more conservative approaches, such as sentinel lymph node biopsy-motivated by the goal of minimizing morbidity without compromising oncologic safety. In this context, preoperative imaging-particularly axillary ultrasonography (AUS), mammography, and magnetic resonance imaging (MRI)-plays an essential role in identifying patients who may safely avoid invasive procedures. However, the growing prevalence of overweight and obesity has introduced new challenges. The accumulation of adipose tissue in the axillary region, as well as fatty infiltration of lymph nodes, may obscure cortical margins and reduce the sensitivity of clinical examination and imaging techniques^(1)^. Understanding how body mass index (BMI) influences the diagnostic accuracy of these methods is therefore of significant clinical and public health relevance, particularly given the high global burden of obesity among women with breast cancer.

In an article published in Radiologia Brasileira, Lyrio et al.^(2)^ present a systematic review and meta-analysis entitled “Evaluation of clinical examination, ultrasonography, mammography, and magnetic resonance imaging for detection of axillary metastases in overweight and obese women with early-stage breast cancer: a systematic review and meta-analysis”. Following the PRISMA-DTA guidelines, the authors synthesized data from nine studies evaluating the diagnostic performance of clinical examination and imaging modalities in overweight and obese patients. Methodological rigor, transparency in study selection, and the use of the QUADAS-2 tool to assess risk of bias are among the strengths of their work^(3)^. The main conclusion-that neither clinical examination nor AUS loses diagnostic performance in overweight or obese women-has practical implications for daily radiological practice. Across studies, AUS remained the most reliable and accessible tool for axillary staging, showing preserved sensitivity and specificity across BMI categories. Although one retrospective cohort study, conducted by Macaione et al.^(4)^, suggested that AUS has a low negative predictive value in obese patients, methodological limitations and nonstandard criteria reduce the generalizability of that finding. The Lyrio et al.^(2)^ review also highlights exploratory evidence that novel mammographic axilla views and MRI may offer complementary information, although both modalities still require validation in larger, standardized cohorts. Despite the limited number of studies specifically designed for BMI-stratified analysis-and the heterogeneity that precluded data pooling (I^2^ = 73%)-the convergence of results strengthens the conclusion that current imaging protocols remain effective regardless of BMI.

As the first systematic review focusing exclusively on this underrepresented subgroup, this study fills a relevant gap in the literature. Its limitations, however, must be acknowledged: most included studies were retrospective, few provided complete 2 x 2 diagnostic tables stratified by BMI, and the AUS criteria for nodal abnormality varied widely. These factors highlight the urgent need for standardization of axillary imaging parameters-such as cortical thickness, shape ratio, and hilum visibility-to enable valid comparisons across populations and imaging centers. Nevertheless, by consolidating evidence that was previously fragmented, this meta-analysis supports maintaining current imaging strategies for overweight and obese women and cautions clinicians to interpret negative AUS findings with careful clinical correlation rather than routine modification of diagnostic protocols.

Looking ahead, this synthesis opens avenues for a new generation of research aimed at personalizing axillary evaluation. Prospective, multicenter studies stratified by BMI are essential to confirm these findings and to clarify whether obesity-related anatomical and inflammatory changes subtly influence diagnostic thresholds. Advances such as contrast-enhanced ultrasound, diffusion-weighted MRI, and radiomics-based texture analysis may soon allow quantification of microscopic features associated with nodal metastasis, potentially mitigating the masking effects of fatty tissue. In addition, the integration of artificial intelligence and deep-learning algorithms offers promise for improving lesion detection and reproducibility in obese patients, fostering a more equitable and precise radiological practice.

In summary, the work of Lyrio et al.^(2)^ supports the idea that accurate axillary evaluation is attainable across BMI categories, underscoring the robustness of physical examination and AUS as cornerstones of axillary staging. As the prevalence of obesity continues to rise worldwide, radiologists must remain cognizant of its potential impact but also confident in the evidence showing that, when performed with a standardized technique, the diagnostic performance of axillary imaging remains reliable. This study contributes not only to clinical reassurance but also to the ongoing refinement of imaging standards in breast cancer care.
